# Polyamide-Laccase Nanofiber Membrane for Degradation of Endocrine-Disrupting Bisphenol A, 17α-ethinylestradiol, and Triclosan

**DOI:** 10.3390/polym11101560

**Published:** 2019-09-25

**Authors:** Milena Maryskova, Miroslava Rysova, Vit Novotny, Alena Sevcu

**Affiliations:** 1 Institute for Nanomaterials, Advanced Technologies and Innovation, Technical University of Liberec, Bendlova 1409/7, 46117 Liberec, Czech Republic; miroslava.rysova@tul.cz (M.R.); vit.novotny@tul.cz (V.N.); 2 Faculty of Mechatronics, Informatics and Interdisciplinary Studies, Technical University of Liberec, Studentska 1402/2, 46117 Liberec, Czech Republic

**Keywords:** laccase, polyamide 6, nanofibers, wastewater treatment, endocrine disrupting chemicals

## Abstract

Contamination of potable water by endocrine disrupting chemicals (EDCs) is a growing problem worldwide. One of the possible treatments is the utilization of laccase enzyme catalyzing oxidation of phenolic structures of EDC when anchored in a polymeric nanofiber membrane. Previous studies failed to develop a membrane with a sufficiently active enzyme, or the immobilization process was too complicated and time-consuming. Here, we established an elegant method for immobilizing *Trametes versicolor* laccase onto polyamide 6 nanofibers (PA6-laccase) via adsorption and glutaraldehyde crosslinking, promoting high enzyme activity and easier applicability in water treatment technology. This simple and inexpensive immobilization ensures both repeated use, with over 88% of initial activity retained after five ABTS catalytic cycles, and enhanced storage stability. PA6-laccase was highly effective in degrading a 50-µM EDC mixture, with only 7% of bisphenol A, 2% of 17α-ethinylestradiol, and 30% of triclosan remaining after a 24-h catalytic process. The PA6-laccase membrane can lead to the improvement of novel technologies for controlling of EDC contamination in potable water.

## 1. Introduction

Over recent decades, the list of known environmental pollutants has been widened by chemical compounds occurring at very low concentrations. Most of these only became detectable following significant progress in available analytical methods. These emerging micropollutants represent a new and, as yet, insufficiently explored form of toxicity, not least due to their remarkable persistence in the aquatic environment and their ability to bioaccumulate. Many of these compounds are capable of short- and long-term toxicity, disruption to the endocrine system, or contribute to the antibiotic resistance of microorganisms [1].

Conventional wastewater treatment methods are insufficient for complete reduction of some pollutants, especially endocrine disrupting chemicals (EDCs). Wastewater treatment plants are only capable of removing or transforming a limited amount of these compounds, either through sorption onto activated sludge or common degradation processes [2]. While progressive technologies such as photocatalysis, UV oxidation, ozonation, Fenton’s reagent, super-critical water oxidation, or ultrasound and ionizing radiation appear to be more effective in removing some EDCs [3,4], most of these approaches require high energy and reagent input. The future strategy of EDCs treatment in the EU, according to the Directive 2013/39/EU of the European Parliament, is based only on two alternative processes: ozonation and treatment with powdered activated carbon [5]. Ozonation is potentially hazardous due to toxicity associated with the formation of possible harmful by-products (e.g., suspected human carcinogen bromate when bromine appears in water) [6]. Activated carbon possesses high adsorption capacity of organic matter in a combination of small particle size and prolonged contact time. On the other hand, used carbon needs to be separated and sent for destruction and re-activation through incineration [7]. Alternative technologies involve nanofiltration, reverse osmosis, and enzymatic treatment. From this perspective, nanofibers represent the most promising material [8,9].

Laccase, an oxidoreductase, shows great promise for use in wastewater treatment due to its sufficient substrate specificity, short reaction time, and low energy consumption [10]. Pure laccase is costly, mainly due to the complicated purification process needed and low commercial demand. As such, it makes little sense to target the application of laccase in its soluble form at present. Instead, it may prove more cost-effective to develop methods for enzyme immobilization, thereby ensuring a long-term effect and enhanced durability in the aquatic environment.

Numerous previous studies have addressed enzyme immobilization, including immobilization of oxidoreductases for wastewater treatment. Most of these focus on laccase as the optimal candidate [11,12,13,14], with peroxidase [15,16,17,18] and fungal tyrosinase [19,20,21] less often chosen. More recent studies have also described immobilization of two enzymes synergistically, thereby combining their efficiencies [22,23,24,25]. Of the available immobilization techniques using different forms of the matrix (e.g., nanoparticles, beads, foams, nanofibers, mats), nanofibers appear to be the most promising for wastewater treatment as they can be used to form safe and easily handled macroscopic mats with a high specific surface area.

Cost-effectiveness and safety of the final nanofiber-laccase membrane are required to be applicable in the water treatment technology. Polyamide 6 (PA6) is probably the cheapest electrospun material with adequate mechanical properties, high stability, and safety. In our previous study, we attempted to develop nanofibers made of PA6/chitosan with bovine serum albumin or hexamethylenediamine as spacers and glutaraldehyde as a crosslinker to reach the highest possible laccase loading and activity [11]. Although the final material was promising due to high enzyme activity and the presence of popular chitosan, the immobilization process took a long time and required too many activation steps, which may prevent its commercialization.

Therefore, our next step was to develop an easier, less laborious method for laccase immobilization onto PA6 nanofibers, achieving the same or better properties as the PA6/chitosan-laccase. The resultant PA6-laccase membrane was tested for its applicability for EDC degradation using a mixture of three well-known EDCs, bisphenol A, 17α-ethinylestradiol, and triclosan.

## 2. Material and Methods

### 2.1. Reagents

Laccase from *Trametes versicolor* (>10 U/mg, powder), 2,2’-azino-bis(3-ethylbenzothiazoline-6-sulphonic acid) (ABTS; >98%), bisphenol A (BPA; ≥99%), 17α-ethynylestradiol (EE2; ≥98%), triclosan (TCS; analytical standard), and glutaraldehyde (GA; Grade II, 25% in H_2_O) were all obtained from Sigma-Aldrich (St. Louis, MO, USA). Pelleted PA6 (B24, M_w_ 37000 g/mol) was obtained from BASF (Ludwigshafen, Germany). All other reagents used were of analytical grade.

### 2.2. Electrospinning of the Nanofiber Matrix

PA6 pellets were dissolved in a mixture of formic acid and acetic acid (2:1; *v*/*v*) to prepare a 12% wt. solution. The nanofibers were then prepared by electrospinning, using Nanospider™ NS 1WS500U equipment (Elmarco, Czech Republic) with a voltage of −20/60 kV and distance between electrodes set at 180 mm. The surface density of prepared nanofiber layers was controlled by adjusting speed of collecting material and by a number of electrospun layers. Specifically, the speed for fabrication nanofibers with 1.5 g/m^2^ was 26 mm/min, and for 3 g/m^2^ was 15 mm/min. Both nanofibers with 5 g/m^2^ and 8 g/m^2^ were prepared from two layers, and the speed was adjusted to 30 mm/min and 15 mm/min, respectively.

### 2.3. Nanofiber Characterization

Images of pristine PA6 nanofibers and nanofibers with immobilized laccase (PA6-laccase) were obtained using SC7620 sputter coater (Quorum Technologies, Lewes, UK) with 10 nm gold layers, Carl Zeiss ULTRA Plus (Zeiss, Oberkochen, Germany) and VEGA3 Tescan (Tescan, Czech Republic) scanning electron microscopes (SEM). Subsequently, the SEM images were analyzed using VEGA TC software for assessing average fiber diameter (obtained by averaging the values of 100 individual measurements).

### 2.4. Enzyme Immobilization

*T. versicolor* laccase was immobilized onto the PA6 nanofibers via adsorption followed by GA crosslinking. A range of parameters, including nanofiber matrix surface density, enzyme solution volume, buffer concentration, and pH, adsorption and crosslinking time, and GA concentration, were examined to establish the most effective immobilization method. Our preliminary experiments identified the optimal immobilization process temperature as 4 °C and the most convenient mode of agitation providing uniform enzyme solution distribution as orbital shaking at 150 rpm. After each immobilization process, the samples were washed with citrate-phosphate buffer (McIlvaine’s buffer) at pH 3 until no laccase activity was detected in the washings, following which the activity of the immobilized laccase was determined.

#### 2.4.1. Influence of Nanofiber Surface Density on the Immobilization Process

Four types of PA6 nanofiber sheets with different surface densities were prepared by adjusting the speed of the electrospinning process. The finest sheet had a surface density of 1.5 g/m^2^ and an average fiber diameter of 79.3 ± 19.8 nm. Subsequent sheets had a surface density of ca. 3 g/m^2^ and a fiber diameter of 87.9 ± 14.7 nm, ca. 5 g/m^2^ and 109.4 ± 19.1 nm, and 8 g/m^2^ and 100.4 ± 23.8 nm.

To evaluate the most suitable nanofiber sheet, circular PA6 samples (diameter 1.5 cm) were immersed into 500 µL of 2 mg/mL laccase solution in 50% McIlvaine’s buffer at pH 3 and shaken for 15 h at 4 °C and 150 rpm. GA was then added to achieve a final concentration of 2.5% (*v*/*v*), and the mixture was shaken for a further five hours under the same conditions. Finally, the samples were washed thoroughly and enzyme activity measured as described in Section 2.4 and Section 2.5.

#### 2.4.2. Influence of Laccase Solution Volume on the Immobilization Process

PA6 samples were immersed into different volumes of 2 mg/mL laccase solution (250 µL, 300 µL, and 500 µL) and incubated according to the procedure described in Section 2.4.1. As the optimal volume appeared to depend on the geometry of the vessel used for immobilization, type of agitation, and sorption of the supporting material, we also examined the optimal vessel type and form of agitation.

#### 2.4.3. Effect of Buffer Concentration on the Immobilization Process

Undiluted (100%) McIlvaine’s buffer was prepared from a mixture of 200 mM disodium hydrogen phosphate and 100 mM citric acid at appropriate ratios that depended on the required pH [26]. Subsequently, the buffer was diluted with ultrapure water to produce a 20% and 50% solution. All three concentrations (20, 50, and 100%) were then used for laccase immobilization. All other parameters of the immobilization process were identical to those described in Section 2.4.2 (i.e., 300 µL of 2mg/mL laccase solution, pH 3).

#### 2.4.4. Effect of Time, pH and GA Concentration on the Immobilization Process

Six different combinations of adsorption time (3, 5, 15, and 24 h) and crosslinking time (3, 5, and 8 h) were used to determine the optimal immobilization duration. The optimal pH for immobilization was evaluated using 20% McIlvaine’s buffer with a pH of 3, 4, 5, 6, and 7. Finally, we tested the GA crosslinker at concentrations of 0.5, 1, 2.5, and 5% (*v*/*v*).

### 2.5. Enzyme Activity Assay

Laccase catalytic activity was measured at 420 nm (ε = 36 mM^−1^·cm^−1^) at room temperature according to Hassani et al. [27] and Maryšková et al. [11] using a BioTech Synergy HTX microplate reader (BioTech Instruments Inc., Winooski, VT, USA). The reaction was performed in McIlvaine’s buffer containing 20 µL of the stock enzyme solution (diluted with 160 µL of buffer) and the same volume of 0.5 mM ABTS. The activity of the immobilized enzyme was measured by adding the sample to 3.6 mL of McIlvaine’s buffer (pH 3) and adding 400 µL of 0.5 mM ABTS. Samples were taken at selected time intervals and measured under the conditions described above.

### 2.6. Storage Stability and Reusability of PA6-Laccase

PA6-laccase was incubated in pH 6 20% buffer at 4 °C to assess storage stability. Two replicate samples were taken at selected time points (13, 20, and 30 days), and their enzyme activity was measured. Free laccase solution was stored under the same conditions for comparison with the immobilized laccase.

Reusability of the PA6-laccase nanofibers was determined by measuring enzyme activity over several catalytic cycles using ABTS as a substrate, as described in Section 2.5. The samples were removed from the ABTS mixture and thoroughly washed with fresh buffer at pH 3 after each catalytic cycle.

### 2.7. Degradation of BPA, EE2, and TCS

The degradation efficiency of free and immobilized laccase was determined by decreasing the concentration of micropollutants over time. One sample of PA6-laccase, PA6 (blank) or 25 µL of the enzyme stock solution (2 mg of enzyme per 1 mL of ultrapure water) were added to glass vials containing 5 mL of a mixture of BPA, EE2, and TCS (50 µM) in ultrapure water containing 30% methanol. Over the selected time intervals, 70 µL of the mixture supernatant was collected into vials containing 65 µL of deionized water and 5 µL of 10% sodium azide, thereby preventing further EDC degradation if some of the enzymes had been collected with the supernatant [28]. Each experiment was performed in duplicate, and the results presented as the mean value ± standard deviation.

EDC (BPA, EE2, TCS) degradation by laccase and PA6-laccase was measured using a Dionex Ultimate 3000 high-pressure liquid chromatograph (HPLC, Thermo Fisher Scientific, Waltham, MA, USA), with an LPG-3400SD quaternary gradient pump, a SR-3000 solvent rack, a WPS-3000TSC autosampler, a TCC-3000SD column compartment, a DAD-3000 detector, and a Phenomenex Kinetex F5 core-shell column (Torrance, CA, USA, length 150 mm, internal diameter 4.6 mm). The aqueous component of the mobile phase consisted of 10 mM phosphoric acid in 5% (*v*/*v*) acetonitrile, while the organic component consisted of 10 mM phosphoric acid in a mixture of 90% acetonitrile and 10% methanol (*v*/*v*). A set of linear gradients were produced, starting with the proportion of the organic component in the mobile phase at 10 %. At 0.7 min the proportion had increased to 15%, at 1.7 min it was 25%, at 3 min 35%, at 4.2 min 40%, at 5.9 min 50%, and at 7.6 min 70%. The proportion of the organic component reached 80% at 8.8 min and from this point up to 9.3 min, the composition of the mobile phase returned to the starting conditions. The chromatogram for each sample was recorded for 12.65 min, with an injection volume of 20 µL, a flow rate of 1.5 mL/min, and column temperature kept at 40 °C. Chromatograms were recorded at wavelengths of 200, 227, 278, and 285 nm, with a sampling rate of 2 Hz.

## 3. Results and Discussion

### 3.1. Nanofiber Matrix Morphology

**Figure 1:**
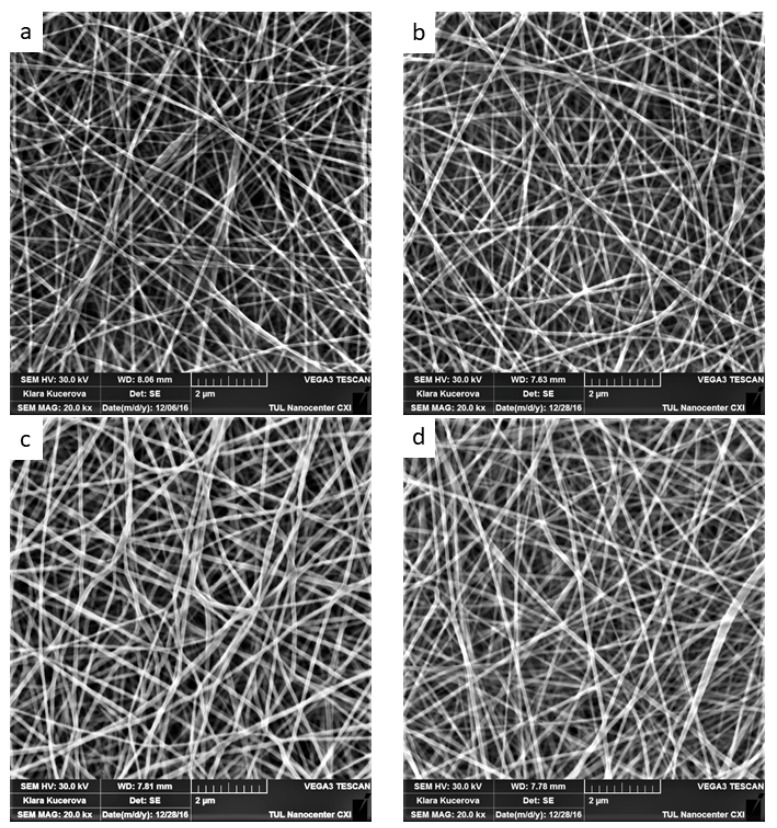
Pristine polyamide 6 nanofibers with surface densities of (**a**) 1.5 g/m^2^, (**b**) 3 g/m^2^, (**c**) 5 g/m^2^, and (**d**) 8 g/m^2^. Images were obtained using Carl Zeiss ULTRA Plus scanning electron microscope (Zeiss, Germany) at a magnification of 20k×.

Support material morphology is an important parameter affecting the enzyme immobilization process. Nanofiber materials are mainly characterized by average fiber diameter and surface density, the combination of these two parameters influencing specific surface area and the immobilization capacity of the matrix [29]. As all our PA6 nanofiber sheets were produced under similar electrospinning conditions, the average fiber diameter was similar for all the nanofiber samples at 79.3 to 109.4 nm (Figure 1a–d). In comparison, the nanofiber materials used in previous studies all consisted of thicker fibers, with typical diameters ranging from 150 to 500 nm [30,31,32]. As such, our PA6 nanofiber sheets had a greater specific surface area for laccase immobilization.

**Figure 2:**
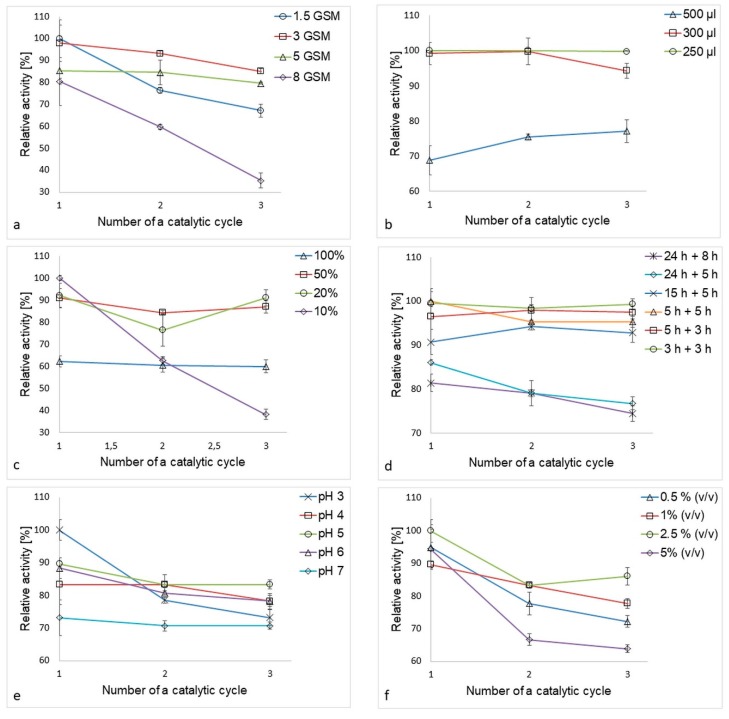
Parameters influencing laccase immobilization—(**a**) influence of PA6 nanofiber matrix surface density, (**b**) influence of enzyme solution volume, (**c**) buffer concentration, (**d**) adsorption and crosslinking time, (**e**) pH, and (**f**) GA concentration.

Despite surface density being an important parameter influencing enzyme loading, substrate/product diffusion, and nanofiber sheet mechanical properties, most studies focus solely on the physico-chemical nature of the immobilization process and tend to ignore the “usability” of the product under actual water treatment conditions [31,33,34,35]. In our study, highest activity levels recorded for immobilized laccase, and the most effective reuse within three ABTS oxidation catalytic cycles, was achieved by PA6-laccase nanofibers with a density of 3 g/m^2^ (Figure 2a). Unfortunately, these nanofibers were not easy to handle due to their poor mechanical properties, a tendency to tear, and deteriorative wettability compared to nanofibers of higher surface density. As such, PA6 nanofibers with a surface density of 5 g/m^2^ were chosen for enzyme immobilization, despite displaying 13 % less enzymatic activity than 3 g/m^2^ PA6 nanofibers.

### 3.2. Optimal Volume of Laccase Solution

Were pristine PA6 nanofibers to be used for enzyme adsorption, it can be assumed that there would be insufficient functional groups to bind the enzyme selectively during the adsorption process. Thus, the volume of enzyme solution will have a strong effect on adsorption efficiency by forcing the liquid to fully adsorb into the nanofiber structure, and as such, the lowest volume ensuring full wetting of the carrier would be most desirable. With insufficient agitation, however, enzyme molecules tend to agglomerate and form sediment. Thus, the volume selected must enable satisfactory enzyme flux in the solution. The lowest volume of laccase solution tested required a very small vessel (0.5 mL vial), which required a higher agitation speed (around 220 rpm) to ensure sufficient motion in the solution. In comparison, a 24-well non-treated polystyrene microplate was able to accommodate PA6 nanofiber samples with a diameter of 1.5 cm, meaning that the samples remained flat throughout the immobilization process. Further, though a slightly higher liquid volume was required (>300 µL), such a vessel allowed easy monitoring of nanofiber immersion level and required a lower agitation speed (<150 rpm). While the best results were obtained using 250 µL of enzyme solution (Figure 2b), such a low volume required a special vessel in ensure complete immersion of the nanofiber sample. Hence, we recommend using 300 µL when using a 24-well plate for immobilization of 1.5 cm nanofiber samples as this encourages full adsorption of the enzyme solution into the nanofibers and ensures that most of the enzyme molecules are in contact with the matrix.

### 3.3. Optimal Buffer Concentration

McIlvaine’s buffer has been used for laccase immobilization and activity assays in numerous previous studies [14,36,37,38]. However, none of these have addressed the optimization of the buffer concentration. Ionic strength is an important parameter influencing both enzyme solubility and the charge of the free functional groups on the enzyme molecules and nanofiber matrix, which in turn enables the development of electrostatic protein–protein and protein–matrix interactions. Generally speaking, lyophilized enzyme powder requires a buffer of sufficient ionic strength to fully solubilize and separate clusters formed by enzyme oligomers [39].

In this study, the clearest influence of ionic strength on the immobilization process was observed between 100% and 10% buffer (Figure 2c). While low ionic strength buffer increased the amount of laccase immobilized, it did not allow for a sufficiently strong interaction between the carrier, enzyme, and GA molecules formed during adsorption and crosslinking, resulting in immobilized laccase of poor stability. In comparison, the highest buffer concentration promoted the formation of strong interactions, resulting in increased enzyme stability. However, possibly due to conformational changes caused by the formation of strong bonds, this concentration yielded the lowest activity of all the samples tested. As a compromise, we were able to achieve both reasonable activity and stability when using 20% and 50% McIlvaine’s buffer, though, from an economic point of view, the less concentrated buffer would be preferable.

### 3.4. Optimal Time for Adsorption and Crosslinking

Time taken for laccase adsorption and GA crosslinking has a clear influence on the activity and stability of immobilized laccase. Surprisingly, the longest time (24 + 8 h) for both adsorption and crosslinking gave the worst results, with lowest immobilized enzyme activity and stability (Figure 2d), probably as the catalyst was damaged through long agitation. Enzyme activity and reuse increased at shorter times. Optimal results were obtained after three hours adsorption and three hours GA crosslinking. This total of six hours compares well with previous studies, where the most common time for laccase adsorption was up to two hours, though adsorption usually took place overnight [31,40].

### 3.5. Optimal pH and GA Concentration

One of the most important parameters influencing immobilization is pH. In our study, the most suitable pH for immobilization of laccase onto PA6 nanofibers was evaluated as pH 5, although the highest stability over three ABTS oxidation catalytic cycles was recorded at pH 7 (Figure 2e). In previous studies [30,31,32,40,41], a similar pH (4–5.5) was also considered as optimal, probably as laccase is best able to maintain its functional conformation when attached to a matrix.

Of the four GA concentrations tested (0.5, 1, 2.5, and 5% *v*/*v*), the highest operational stability was observed at the highest GA concentration (5% *v*/*v*). Surprisingly, the optimum concentration turned out to be 2.5% *v*/*v*, providing both the highest activity and best reuse of immobilized laccase. We hypothesize that, at this concentration, larger enzyme clusters were formed outside the matrix area, which decreased the number of bonds between the enzyme and the support (Figure 2f).

GA has long been favored as a crosslinking agent for enzyme immobilization. In a number of cases, it has been used for matrix stabilization [42,43] or functionalization of the supporting material [30,44,45,46], the optimal GA concentration in such cases varying between 1–4% *v*/*v*. In most cases, however, GA has been used as a bifunctional agent for the introduction of aldehyde groups to the matrix surface prior to enzyme attachment [47,48,49,50]. However, this type of functionalization requires the presence of free primary amino groups on the matrix. In comparison, our method does not require the matrix to have such a chemical composition and, as such, provides much greater freedom in the selection of materials for enzyme immobilization.

### 3.6. Summary of the Optimal Immobilization Process

**Figure 3:**
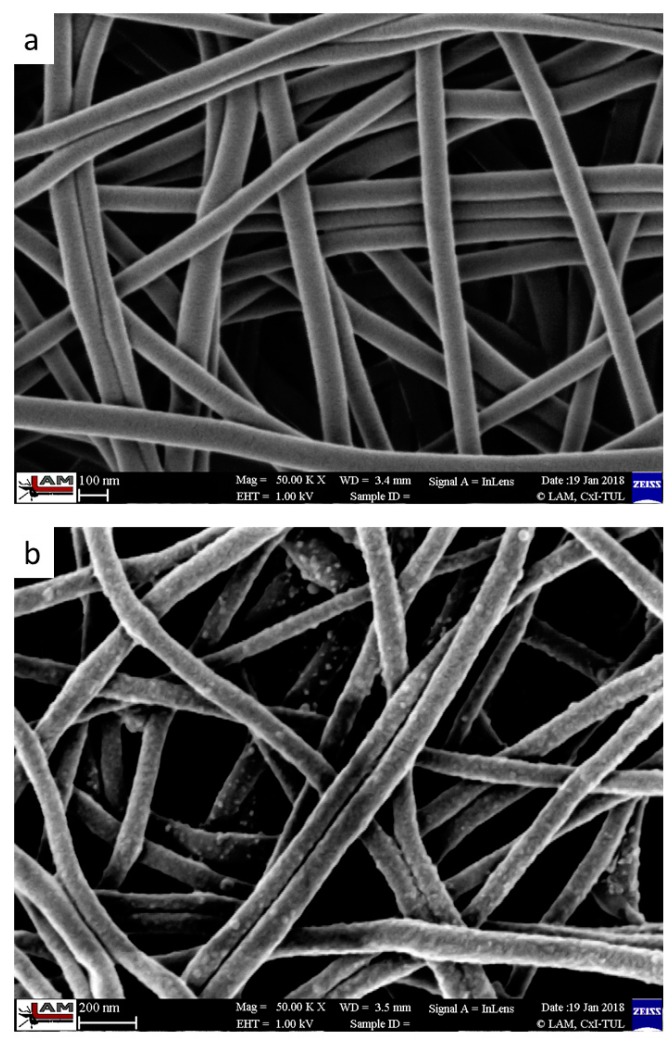
Structure of (**a**) pristine PA6, and (**b**) nanofibers with immobilized laccase (PA6–laccase). Images of pristine PA6 nanofibers and nanofibers with immobilized laccase were obtained using a Carl Zeiss ULTRA Plus scanning electron microscope (Zeiss, Germany) at a magnification of 50k×.

Overall, our results suggest an optimal average PA6 nanofiber diameter of 105 ± 19.1 nm, with a surface density of 5 g/m^2^ (Figure 3). Under these conditions, the nanofibers display excellent mechanical properties, ease of handling, repeatability, low cost, and homogenous surface density, thereby providing perfect conditions for laccase immobilization [51]. We immobilized *T. versicolor* laccase onto PA6 nanofibers via adsorption followed by GA crosslinking. The optimal immobilization process required the PA6 nanofiber samples (~1 mg each) to be submerged separately into 300 µL of 2 mg/mL laccase solution in 20% buffer at pH 5 and shaken at 4 °C in an orbital shaker at 150 rpm for three hours. GA was then added to achieve a final concentration of 2.5 % *v*/*v*, and the samples were shaken in the immobilization solution for a further three hours. Finally, the samples were washed with McIlvaine’s buffer at pH 3. Pristine PA6 nanofibers display a homogenous structure and smooth surface (Figure 3a), while PA6-laccase displays a grainy surface formed by cross-linked laccase clusters strongly attached to the nanofibers, even after thorough washing (Figure 3b).

In comparison with our previous study [11], the immobilization technique using PA6 as a matrix is more straightforward. While before, we aimed for the highest possible enzyme loading onto PA6/chitosan material, here we focused on the least expensive and time-consuming method. First, PA6 nanofibers are more easily manufactured and much cheaper compared to PA6/chitosan blend. Second, we succeeded in reducing and simplifying the crosslinking process to a single reaction with GA, instead of double GA crosslinking and single bovine serum albumin or hexamethylendiamine activation. The whole procedure was in total shortened by 21 h. Besides that, the new laccase immobilization step required approximately three times lower bath volume. Although the PA6-laccase membrane reached slightly lower catalytic activity than PA6/chitosan-laccase, this drawback was balanced by 50% higher reuse activity.

### 3.7. Storage Stability and Reuse

**Figure 4:**
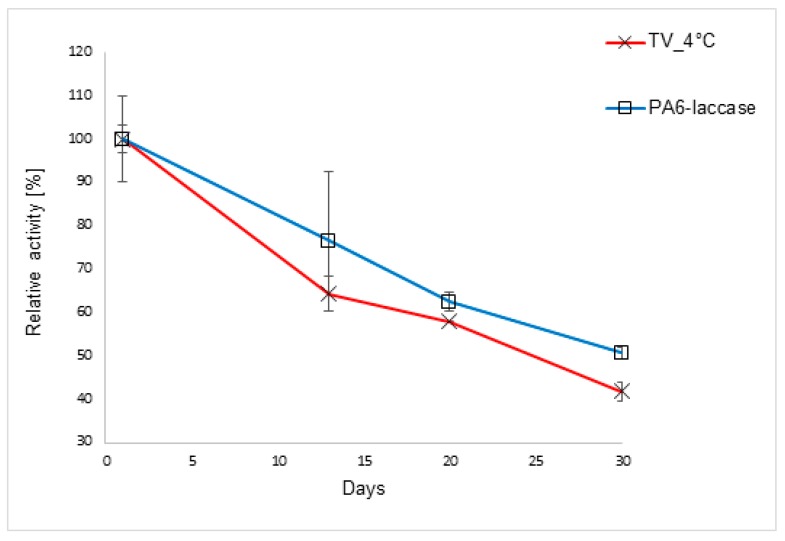
Storage stability of free and immobilized laccase. PA6 nanofibers loaded with immobilized laccase were incubated in 20% buffer (pH 6) at 4 °C. Free laccase solution was stored under the same conditions for comparison with the immobilized laccase.

The storage stability of free and immobilized laccase was tested by assessing activity after 13, 20, and 30 days of storage at 4 °C. Use of pH 6 McIlvaine’s buffer and a temperature of 4 °C provided optimal conditions for the preservation of enzyme activity, with laccase retaining 42% of its initial activity. PA6-laccase displayed similar storage stability to the free enzyme, with 50% of activity retained after 30 days (Figure 4).

In previous studies, laccase immobilized onto fibrous polymer-grafted polypropylene chloride film preserved around 57% of initial activity after 30 days storage in a pH 5.5 buffer at 4 °C [52], while laccase immobilized onto a PVDF membrane retained 43% of initial activity after 36 days of storage in pH 4 McIlvaine’s buffer at 4 °C [14]. In an exceptional case, laccase immobilized onto carbon nanotubes retained 80% of initial activity under similar storage conditions, while free laccase retained a similar activity level as that in our study [48]. Thus, it appears that particle or nanoparticle carriers are better in preserving the enzyme activity, probably due to their favorable pore size distribution compared to fibrous or nanofibrous structures. However, these nanomaterials cannot compete with the PA6 nanofiber matrix regarding safety, ease of handling, and applicability under actual wastewater treatment conditions. PA6-laccase retained 88% of its initial activity after five ABTS oxidation catalytic cycles; a very promising result compared with the study of Xu et al. [53], where laccase covalently immobilized onto carbon nanotubes retained ca. 80% activity after five ABTS transformation cycles, and [54], where *T. versicolor* laccase immobilized onto magnetic bimodal mesoporous carbon retained 70% activity after five cycles.

### 3.8. Degradation of BPA, EE2, and TCS

**Figure 5:**
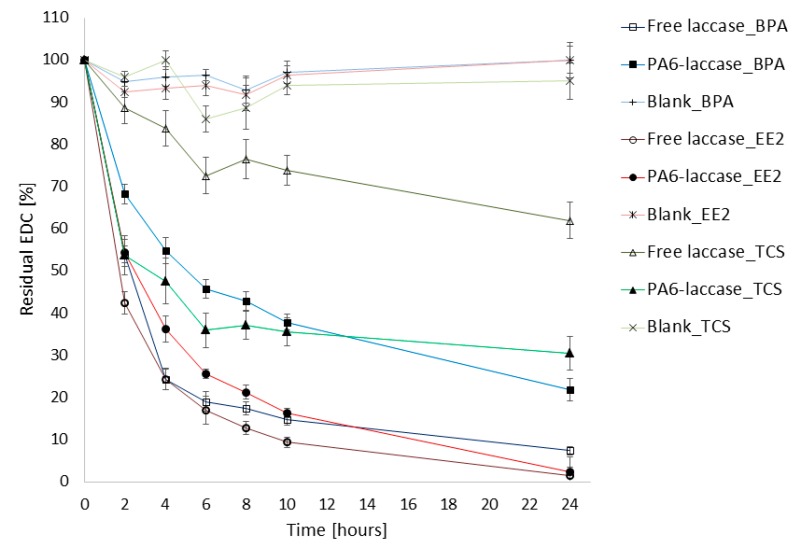
Comparison of degradation efficiency of free and PA6 immobilized laccase against BPA, EE2, and TCS.

We tested the degradation activity of laccase and PA6-laccase against a 50 µM BPA, EE2, and TCS micropollutant mixture. Our PA6-laccase displayed activity of 0.03 U, similar to that of free laccase in 25 µL of the enzyme solution. PA6-laccase removal efficiency of BPA was lower than that of free laccase (22% remaining after 24 h), though the removal profile was similar to that of free laccase with EE2. PA6-laccase was notably more efficient in TCS reduction within 24 h of incubation (ca. 70% decrease compared with 38% for free laccase; Figure 5). In comparison, a blank sample containing only PA6 nanofibers did not adsorb any EDCs over the 24 h.

Degradation of EDCs using immobilized laccase has been the subject of a number of studies [55,56]. *Cerrena unicolor* laccase (12 U/5 mL) captured on porous silica beads, for example, has been shown to eliminate around 90% of BPA (50 µM) after 60 min of incubation [57]. Application of nanoparticles for enzyme immobilization has recently been criticized due to issues connected with their commercial application, such as their tendency to agglomerate and the need to separate them from treated water [58,59]. The mechanical properties of PA6, on the other hand, showed no signs of damage over 30 days of storage. Hence, PA6 has a major advantage over particle- or nanoparticle-matrices in that their macroscopic and compact form allows them to be handled as textiles, providing great potential for their use as stable filters in water treatment processes.

## 4. Conclusions

The effectiveness of PA6-laccase for EDC degradation, along with its capacity for repeated use and enhanced storage stability, predetermine this material for real application in advanced wastewater treatment technology. Currently, the cost-effectiveness of immobilized enzymes for water treatment depends on the price of commercially available products and the immobilization method used. Recently, the most popular materials used as enzyme carriers were alginate and similar gels, nanocellulose, zeolites, or bentonite. Compared to these materials, pristine PA6 nanofibers do not require any further chemical modifications, are low-cost, easily manufactured, safe to handle, and possess exceptional mechanical properties beside other electrospun polymers. In this study, we developed a simple and reproducible technique for laccase immobilization onto PA6 nanofibers using only GA as a reagent. Our methodology proved simple and inexpensive thanks to short adsorption and crosslinking time and the use of easily fabricated support and readily available GA. All these features can facilitate inclusion into already existing filtration units without a risk that the matrix-enzyme system will leak into the effluent. Furthermore, we tested degradation efficiency toward the elimination of highly concentrated solution of environmentally hazardous endocrine disruptors BPA, EE2 and TCS. The PA6-laccase system was successful, and the immobilized laccase reached very similar results compared to the free soluble form. If higher demand increases commercial production of laccase and thus reduces its market price, PA6-laccase can enable a new cost-effective and efficient technology for the elimination of EDCs in potable water.
